# Plant-Based and Hybrid Patties with Healthy Fats and Broccoli Extract Fortification: More Balanced, Environmentally Friendly Alternative to Meat Prototypes?

**DOI:** 10.3390/foods14030472

**Published:** 2025-02-01

**Authors:** Josemi G. Penalver, Maite M. Aldaya, Débora Villaño, Paloma Vírseda, Maria Jose Beriain

**Affiliations:** 1 Institute for Sustainability & Food Chain Innovation (IS-FOOD), Public University of Navarra (UPNA), Jerónimo de Ayanz Building, Arrosadia Campus, 31006 Pamplona, Spain; josemiguel.gonzalez@unavarra.es (J.G.P.); maite.aldaya@unavarra.es (M.M.A.); debora.villano@unavarra.es (D.V.); virseda@unavarra.es (P.V.); 2 Agronomy, Biotechnology and Food Department, Public University of Navarra (UPNA), Arrosadia Campus, 31006 Pamplona, Spain; 3 Science Department, Public University of Navarra (UPNA), Arrosadia Campus, 31006 Pamplona, Spain

**Keywords:** carbon footprint, high hydrostatic pressure, vacuum cooking, hybrid protein, product development

## Abstract

Hybrid and plant-based products are an emerging trend in food science. This study aimed to develop three patty prototypes (meat, hybrid, and plant-based) enhanced with vegetable fat replacement and broccoli extract using a soy allergen-free protein matrix treated with high hydrostatic pressure (HHP) and sous vide cooking to create sustainable and nutritious burger alternatives. The samples were evaluated for microbiological safety, proximal composition, physicochemical properties, sensory characteristics, and carbon footprint. The key findings revealed that the plant-based patties had the smallest carbon footprint (0.12 kg CO_2_e), followed by the hybrid patties (0.87 kg CO_2_e) and the meat patties (1.62 kg CO_2_e). The hybrid patties showed increased hardness, cohesiveness, gumminess, and chewiness compared to the meat patties after sous vide treatment. This improvement likely results from synergies between the meat and plant proteins. Regarding the treatments, in all the samples, the highest hardness was observed after the combined HHP and sous vide treatment, an interesting consideration for future prototypes. Sensory analysis indicated that the plant-based and hybrid samples maintained appealing visual and odour characteristics through the treatments, while the meat patties lost the evaluator’s acceptance. Although further improvements in sensory attributes are needed, hybrid patties offer a promising balance of improved texture and intermediate carbon footprint, making them a viable alternative as sustainable, nutritious patties.

## 1. Introduction

To meet the rising food demand driven by exponential population growth, global agricultural production must increase by 70% by 2050, requiring substantial boosts in both meat and cereal outputs [1]. Achieving this growth in a sustainable way will require the fundamental transformation of food systems, one that minimises the environmental impact, while ensuring equitable access to nutrition [2].

To address this growing demand, the food industry is increasingly focusing on the development of hybrid products that combine meat with plant-based ingredients. These products aim to reduce overall meat consumption, while increasing vegetable intake, addressing both health and sustainability concerns [3,4]. This innovation aligns with a growing societal shift toward plant-based alternatives, driven by the pursuit of more sustainable and environmentally friendly diets [5]. A large number of food innovation studies aim to achieve this shift, without compromising the taste or nutritional value that meat provides in consumers’ diets [3,4], since animal proteins are renowned for their high quality and for providing essential nutrients, such as iron, zinc, and B-complex vitamins [6]. The proportions of meat and plant-based ingredients in hybrid meat products vary, incorporating a range of plant options, such as fruits, vegetables, and legumes, either individually or in combination [7].

When developing hybrid food products, understanding the behaviour of fused food matrices is crucial. Beyond the anticipated differences in macro- and micronutrient compositions, the interactions between plant proteins (with a typical globular, tertiary structure) and animal proteins (which, in the case of meat, are predominantly fibrous) are of particular interest. These interactions can significantly influence the texture, functionality, and nutritional quality of the final product [8]. With this in mind, high hydrostatic pressure (HHP) and vacuum cooking (sous vide, VC) techniques have garnered significant attention for their impact on the physicochemical properties of foods. HHP, in particular, exposes foods to extreme pressures, yielding multiple benefits, enzyme inactivation, enhanced digestibility, and the elimination of microorganisms, all while preserving the nutritional value and the vitamin content. Notably, its denaturing effect on proteins leads to critical changes in their technological properties, further enhancing the product’s performance [9]. Vacuum cooking, on the other hand, enhances both sensory and nutritional qualities by cooking at low, controlled temperatures. This method produces more uniform results when it is applied, while also denaturing the tertiary structure of proteins and minimizing moisture and micronutrient loss during cooking depending on the protein matrix [10].

In the pursuit of enhancing consumers’ nutrition, while minimizing the impact on natural resources, broccoli extract has emerged as a promising local by-product [11]. This extract is rich in glucosinolates, biocompounds naturally found in cruciferous vegetables such as broccoli, with noteworthy properties, including antioxidant and anti-inflammatory effects, and they play a role in modulating carcinogenic activity, increasing the nutritional value that patties can offer to the consumer [12,13]. However, glucosinolates are highly sensitive to external factors like light and heat, making them prone to degradation. This sensitivity necessitates the careful consideration of the effects of various treatments during product processing, as these can significantly influence the quantity and bioavailability of these valuable compounds [14]. For this reason, this type of extract is not commonly used in the food industry. In this research, broccoli extract was encapsulated with a double layer of modified starch. Through this method, the bioactive compounds were stabilised, ensuring their integrity and availability during the cooking process and until the prototype was consumed [15]. Furthermore, according to the recent literature, the application of high-pressure treatments could enhance the functionality of glucosinolates, reinforcing their role as health-benefitting compounds for consumers [16]. To address the sustainable obtention of these biocompounds, research on the utilisation of broccoli crop residues and surpluses has become increasingly important [11,17]. Such studies aim to enhance agricultural practices, optimise food-related industrial processes, and ultimately contribute to improved human nutrition [12,13]. This approach aligns with the present project on sustainable practices by utilizing local resources to promote both health and environmental stewardship.

Recent studies have explored the application of innovative techniques in the development of beef, extruded soybean, and hybrid patties, focusing on the effects of various fats and substitutes on their physicochemical and sensory properties. The use of HHP, VC, and their combination yielded promising outcomes, enhancing both the quality and safety of these hybrid products. These methods confirmed the suitability of hybrid patties for consumption, while preserving their desirable organoleptic characteristics [18,19,20].

In line with the previous product development efforts [21], this study selects pea ingredients as a plant-based alternative to soy due to its gluten-free nature, high digestibility, and richness in proteins, fibre, and essential amino acids [22], while maintaining good technological properties [23,24], making it an excellent choice for a wide range of consumers, especially those with dietary restrictions or sensitivities [25]. Pea protein is particularly noted for its mild sensory profile, making it an appealing option in the creation of plant-based products [26]. In addition, broccoli extract will be incorporated into the developed patties, which will enhance the nutritional properties, incorporating bioactive compounds. The prototypes developed will be assessed not only for their organoleptic and psychochemical qualities, but also through a carbon footprint assessment. This parallel study aims to provide quantifiable evidence of the environmental benefits associated with this innovative product.

## 2. Materials and Methods

### 2.1. Ingredients and Patty Preparation

This study focused on producing patties using three different formulations: a meat-based product, a plant-based product, and a mixture of both, a hybrid product. The meat (*Biceps femoris*) used in both the meat-based and hybrid patties was sourced from Ternera de Navarra, veal with Protected Geographical Indication, raised through extensive cattle farming methods. The plant-based ingredient was pea flour marketed by Molendum Ingredients. This product is made from the milling of *Pisum sativum*, which is industrially cleaned, gluten-free, and soy-free. Peas are a locally sourced product from Navarra, reducing the transport requirements and potentially making the process more sustainable. This approach aligns with the objectives of the ALISSEC project (Design of healthy and sustainable food and ingredients from the circular economy), a regional project under which the present study was carried out, which aims to encourage Navarrese companies to use peas as a protein source instead of soy. The preparation of the plant-based samples involved hydrating flour with 48.8 g of water per 100 g of raw material, followed by thorough mixing. The hybrid patties were formulated by combining equal portions of the plant-based product (hydrated raw material) and the meat.

Additionally, the prototypes included broccoli extract derived from agro-industrial by-products from the Navarre region, a glucosinolate-rich extract, aimed to enhance prototypes’ health contribution to the consumer’s diet. Using research by Janardhanan et al. [19], the animal fat part was replaced with a hydrogel from olive and linseed oil, reducing the saturated fat content and ensuring the absence of soy allergens. In this sense, soy is commonly used for vegetable fat emulsions to replace animal fat for its textural properties. This hydrogel emulsion was created based on the method described by Poyato et al. [27], with some modifications; xanthan gum was added to minimise synergistic losses in the emulsion.

First, the mixture consisted of an oil phase with 23.82 g of olive oil, 15.88 g of linseed oil, and 0.05 g of polysorbate-80 per 100 g. The mixture was prepared in parallel to the aqueous phase, consisting of 1.49 g of κ-carrageenan, 58.02 g of water, and 0.74 g of xanthan gum per 100 g of mixture. Both phases were heated separately to 70 °C in continuous agitation and kept at that temperature for 10 min. The oil phase was gradually added to the carrageenan–water mixture and homogenised with a mechanical immersion blender at 16,000 rpm for 20 min. After homogenization, the hydrogel emulsion was sealed, allowed to cool to room temperature, and then refrigerated at 4 °C overnight to facilitate polymerization.

According to Janardhanan et al. [19], the patties were prepared by first blending the protein matrix (72.63 g/100 g), with the oil-in-water emulsion (21.3 g/100 g), broccoli extract (4.68 g/100 g), and salt (1.41 g/100 g) using a mixer (Professional Mixer Series 6, KitchenAid™, St. Joseph, MI, USA) until a uniform mixture was achieved. The mixture was then pressed into patties. The patties were subsequently vacuum-packaged in bags using a chamber vacuum machine (C412 Lerica, Venice, Italy). Finally, the samples were stored at 4 °C overnight until they froze, or they were pressurised (HHP ones). The type of sample and percentage of composition according to each ingredient can be found in Table 1.

### 2.2. Experimental Design and Treatments

**Figure 1:**
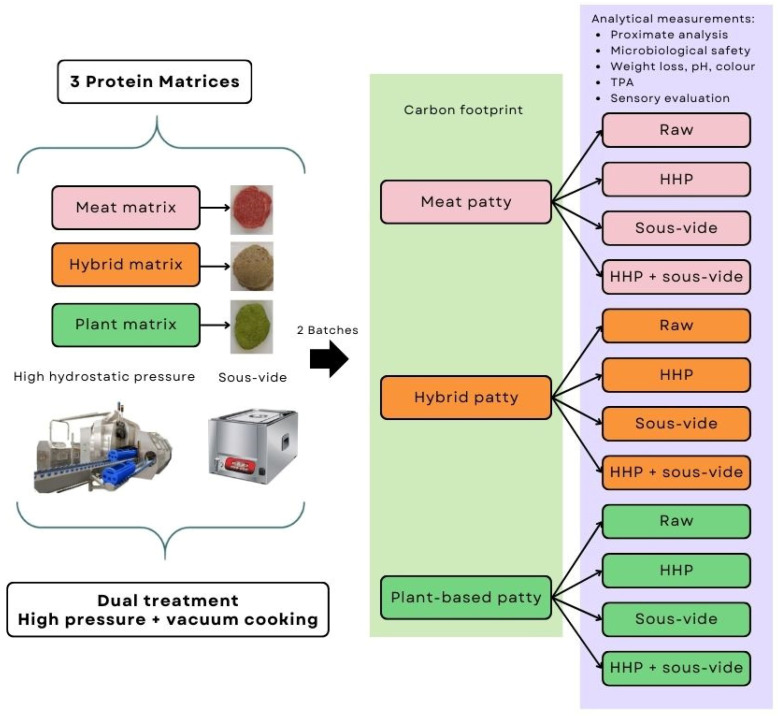
Experimental design flowchart: types of sample, culinary treatment, and batches.

The treatment selected for the patties, following the previous lines of study, was the use of high hydrostatic pressure and the combined use of HHP + vacuum cooking. The whole experiment was carried out in duplicate (two production batches) to ensure the absence of experimental error. A total of 132 patties were prepared in two batches (66 per batch) with 3 different protein matrices (meat, plant-based, and hybrid) and 4 types of culinary treatment, rendering a total of 12 different formulations. Thirty patties were subjected to HHP. Thirty-six patties were subjected to HHP and subsequent VC, and the last thirty-six were only cooked under VC. Finally, 30 patties were analysed without treatment (raw samples). For the formulations that underwent VC treatment, an extra patty was prepared and used to control the temperature of the process. The process is illustrated in the flowchart shown in Figure 1.

Once vacuum-sealed, the HHP samples were placed in a pressure chamber using a containment basket that allowed for free movement of the samples. Within the chamber, they were subjected to high hydrostatic pressure by injecting transmission fluid (in this case, water) and setting the internal chamber pressure to 350 MPa for 10 min. Pressure was maintained consistently throughout the treatment to ensure uniform processing. Each batch of product was treated separately to eliminate variability due to the treatment. Upon completion of the process, pressure was gradually released to avoid abrupt temperature changes or structural damage to the patties. This procedure was carried out using an Idus machine (Idus HPP Systems S.L.U., Noain, Spain) with a 25 L vessel capacity. These time and pressure conditions were selected based on the best results obtained by Janardhanan et al. [28].

The pressurised samples were subjected to vacuum cooking the following day. A baking bath (Orved SV Thermo-Top, Orved S.P.A., Venice, Italy) was used for vacuum cooking at a low temperature. The samples were introduced into the temperature-controlled bath when the water temperature reached 55 °C. The HHP samples were cooked at a temperature of 55 °C for 15 min [19]. Temperature-sensing probes inserted in a reference sample were used to determine the core temperature of the product at all times. Once the core maintained the set temperature for 15 min, the samples were immersed in cold water to stop cooking. The treated samples were stored at 4 °C until further analysis.

### 2.3. Characterization of the Samples

#### 2.3.1. Proximal Analysis of Samples

The different types of patty were analysed in triplicate according to the three protein matrices used for formulations. For the characterization of proximal composition, moisture [29], protein [30], fat [31], and ash content [32] were determined.

#### 2.3.2. Microbiological Testing

The microbiological analysis of the cooked samples was required to ensure that the cooking treatments applied were effective in ensuring their safety. Microbial tests for *Salmonella* species [33], *Listeria monocytogenes* [34], and *Escherichia coli* B-glucuronidase [35] were conducted at Eurofins Análisis Alimentario, Nordeste SL (Spain).

#### 2.3.3. Physicochemical Characterization of Samples: pH, Weight Loss and Colour

##### Determination of pH

The pH of the 12 sample formulations was measured in triplicate at 25 °C [36] using a pH meter (Crison Instruments S.A., Barcelona, Spain).

##### Determination of Weight Loss

Following the calculation recommendations of Murphy et al. [37], the weights of the samples before and after the different treatments (VC, HHP, or the combination of both) were taken. The raw, untreated samples were also characterised for this parameter; their weight was measured after the cooling process in order to have a reference of natural weight losses that may occur without the application of these treatments. Weight loss (or cooking loss when cooked) was calculated using the following formula:

$$Weight\;loss\left(\%\right)=\left[\left(Mb-Ma\right)\times 100\right]/Mb$$

In this formula Mb and Ma represent the weights of the samples before and after treatment, respectively.

##### Determination of Colour

The colour of the sample was characterised using a portable spectrophotometer (Minolta CM-2300d, Konica Minolta Business Technologies Inc., Tokyo, Japan). Analysis was performed with the colour parameters L, a*, and b* using the CIELAB colour space system [38].

#### 2.3.4. Instrumental Texture of Samples

Texture profile analysis (TPA) was performed on the untreated and treated samples with either HHP, VC, or a combination of both (HHP + VC). For this purpose, texture analyser (TA-XT2i, Stable Micro Systems Ltd., Surrey, UK) equipment was used. The data from six consecutive measurements were collected using Exponent Lite version 6.1 software (Stable Micro Systems Ltd., Surrey, UK). The analysis protocol was carried out in accordance with Mittal et al. [39]. From the data recorded in the strength/time profile, the following parameters were studied: hardness, springiness, cohesiveness, gumminess, and chewiness.

#### 2.3.5. Sensory Analysis of Samples

Sensory analysis involved the participation of 16 panellists with previous experience in food sensory characterization; they only had to evaluate the colour and smell of the patties. Prior to testing, the participants in the sensory analysis were informed about the nature of their participation. The participants were explicitly informed of their right to refuse to participate in this study at any time, without any negative consequences or any direct or indirect reprisals for the decision. This research followed the principles and guidelines of the 1964 Helsinki Declaration to ensure the ethical treatment of participants. The Data Controller is from the Public University of Navarra, and the persons authorised to process the data are the Head of the Research Project and members of the Research Team. The participants were able to contact them for queries about the processing of the data. They were free to exercise the rights of access, rectification, deletion, opposition, and portability to the Data Protection Delegate of the Public University of Navarra. Privacy was requested for the food prototypes shown, and the panellists were verbally informed about the protection of personal data. Verbal consent was obtained in consensus with Spanish legislation [40]. The panellists underwent two training sessions of one hour each before evaluation, with the subsequent presentation of the profile sheet before the tasting session [41].

After assigning random values to the samples, one raw sample, one vacuum-cooked, and one that underwent a combination of vacuum cooking and high hydrostatic pressure were presented to the panellist. The tasting session consisted of descriptive analysis of the visual and olfactory attributes. The intensity of these was noted in the form of a non-structured scale with labelled ends. The different parameters analysed were quantified by measuring the distance in centimetres to the panellist’s mark from the left side of the scale from 0 to 15 cm. The parameters were as follows: colour (0 = green/ 15 = pink-brown), aspect (0 = homogeneous/ 15 = non homogeneous), odour (0 = rancid/ 15 = fresh), vegetal odour intensity (0 = low/15 = high), any other odour (free contestation), and global acceptance (0 = I do not like it/ 15 = I like it). Sensory evaluation was carried out in white light-, temperature-, and humidity-controlled booths [42].

### 2.4. Carbon Footprint Characterization

**Figure 2:**
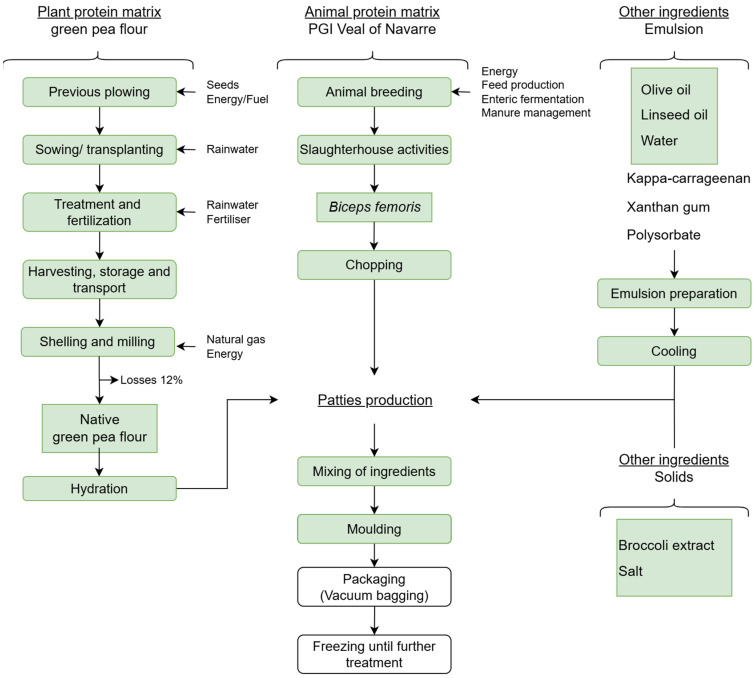
A flowchart of the raw protein materials and patty production with the processes and ingredients included in the carbon footprint (the parts underlined in green).

To support the development of the patty prototypes, carbon footprint analysis was conducted in accordance with relevant international standards [43,44,45]. Analysis focused on greenhouse gas emissions from the ingredients and patty-making processes, which are core to all the three products. This study used a pilot-scale production system. This approach considers all the relevant stages of the ingredients’ life cycle, including the extraction and production of raw materials. This study is focused on the composition and impact of protein matrices on the prototypes. The subsequent processing stages, such as cooking, packaging, distribution, and consumption, were the same for all the prototypes. The system boundaries included direct and indirect carbon emissions, which are illustrated in Figure 2, where the processes and ingredients included in carbon footprint analysis are highlighted in green. The methodology involved the collection of primary data for the production processes of the following local ingredients: meat, pea flour, and glucosinolates-rich broccoli extract (which accounts for approximately 77% of the patty weight). The secondary data were used to obtain specific emission factors (CO_2_e/kg of product) for the remaining non-locally produced ingredients (remaining 23% of the patties weight). Analysis included emissions of carbon dioxide (CO_2_), methane (CH_4_), and nitrous oxide (N_2_O). The results are expressed in terms of carbon dioxide equivalents (CO_2_e) based on their Global Warming Potential [46]. The functional unit of measurement was one patty (150 g) without cooking.

For the calculation of the carbon footprint of the patty-making process, associated emissions were assumed to be similar to the data published by Domínguez-Lacueva et al. [47], who analysed the impact of producing comparable patties with soy-based vegetable protein.

In assessing the carbon footprint of native pea flour, most of the activity data were gathered through surveys directly distributed to green pea farmers (see Supplementary Material, Survey S1: survey distributed to farmers (translated into English)) for agricultural activity in 2021. The specific data regarding the cultivation process and flour production can be found in Text S1 of the Supplementary Material.

The carbon footprint of the veal from Navarra, certified as having Protected Geographical Indication (PGI), was determined, taking into account that it exclusively comprises autochthonous calf breeds, such as Pirenaica, Blonde de Aquitania, Parda Alpina, Charolesa, and their crossbreeds. The methodology and results can be found in Domínguez-Lacueva’s masters dissertation [47].

The approach considered for enhancing the nutritional value of the patties involved using broccoli extract derived from broccoli by-products, which lack market value as they are not generally utilised by the food industry. As a result, only the glucosinolate extraction process impacted the carbon footprint of this ingredient, while the production phase of the broccoli by-products (stem and inflorescence detachment) was not factored into the calculation. The impact data have been directly provided by the researchers responsible for this project, as these are still in the publication process [17].

### 2.5. Statistical Analysis

Statistical analysis was carried out using SPSS statistical software, version 27.0 (IBM Corp., Armonk, NY, USA), to study the results obtained in the different analyses of the patties. The descriptive statistics used were the mean and standard deviation of the measurements taken for each patty. On the one hand, two-factor analysis of variance (ANOVA) was carried out (treatment and protein matrix). On the other hand, the analysis of fixed factor interactions was also conducted whenever feasible using a post-hoc multiple comparison test (Tukey’s analysis with a confidence interval of 95% (*p* < 0.05)). To analyse the results of sensory analysis, visual characterization of the data was conducted using a radar chart.

## 3. Results and Discussion

### 3.1. Proximate Analysis of Patties

Table 2 shows the proximate analysis according to the protein matrices used: meat, hybrid, and plant-based. As expected, clear differences can be found depending on the protein matrix and the applied treatment. Moreover, interactions between the factor’s protein matrix and the treatment applied were observed.

Regarding the fat content, a slight increase was observed in the samples treated sous vide (with and without high-pressure processing, 0.29% and 5.10%, respectively), while the opposite trend was observed in the meat samples (−16.24% and −8.27%, respectively). The plant-based patties show contradictory results on the effect of the treatment on the fat content, as HHP and VC alone seem to increase this parameter (5.95% and 4.44%, respectively), but not when they are combined. The protein levels appeared to decrease when the samples were subjected to sous vide for the plant-based patties (−3.66%), though not when HHP or both the treatments were combined. The hybrid patties, being a mixture of both the sources, were the most affected by the treatment in terms of the protein content (reductions of −7.83% and −3.53% with the VC and HHP treatments, respectively). The hybrid-based samples subjected to either of the treatments showed a lower protein content compared to that of the raw samples; however, this decrease cannot be attributed to a loss of material as the minimum material loss occurred when the sample was taken from its packaging. It should be noted that a Kjeldahl system was used to determine the protein content, where the nitrogen content of the sample was first measured, and a conversion factor of 6.25 was applied to estimate the portion of nitrogen corresponding to the protein [48]. It is hypothesised that the changes in protein content may be due to some leakage of nitrogen (whether protein nitrogen or not) migrating to the plastic surface of the packaging or to the exudates during the application of the thermal and pressure treatments. This migration or loss may be driven by the interaction between the animal and plant proteins and enhanced by the applied treatments, resulting in a lower concentration of nitrogen in the sample, leading to an apparent decrease in the protein content. Although this decrease was minimal, it was noted as significant through the highly sensitive statistical analysis applied. Lastly, the total ash content showed a slight decrease with any of the mentioned treatments compared to that of the untreated sample, being significant only in the hybrid patties. Regarding the differences between the present study and that of Janarhanan et al. [28], it is important to note that in Janarhanan’s study, the plant-based samples were made using extruded flours, rather than natural pea flour as in the present study, which dramatically changes the structure and behaviour of macronutrients, especially protein, in the food matrix [49].

Comparing the groups according to the protein source, the protein content in the meat samples was approximately 17.4%, while plant-based products made mostly from legumes presented a lower average protein percentage (11.8%). As expected, the hybrid samples had intermediate results. The protein level of the legume source reached 19% in the native green pea flour used to develop the prototypes, which is comparable to the protein content of the meat used for the patties. However, this high protein content had to be compromised to enhance the flour’s technological properties. Without prior hydration (which dilutes the protein concentration), it would not have been possible to successfully mix the fat emulsion, glucosinolate-rich broccoli extract, and pea flour. Therefore, the protein levels were lower than desired since generally the meat analogue products on the market provide a higher protein content (20%) than those of the developed prototypes (both, meat, and plant-based samples). However, the recent literature highlights that from a nutritional standpoint, these plant-based food products may not be considered the most suitable “short-term” substitute for meat, mainly because of the great differences found in their micronutrient composition (vitamins, minerals, bioactive components, etc.) [50].

In terms of moisture, pea flour prior to hydration contained 15% moisture, while the meat, as a raw ingredient, had an average of 75.5% [51]. Therefore, despite the pre-hydration of flour, the moisture content of the meat or partially meat-based samples remained notably higher.

Regarding the remaining parameters, the ash content was quite similar across the samples regardless of the protein matrix used. The fat content was also consistent, which makes sense given that the vegetal fat replacement was supplied in the same quantities for each sample.

### 3.2. Microbiological Testing

The results of microbial counts in the hybrid samples cooked (55 °C, 15 min) with and without high-pressure treatment (350 MPa, 10 min) are shown hereafter. All the counts remained within acceptable limits according to the Commission Regulation (EC) No 2073/2005 of 15 November 2005 on microbiological criteria for foodstuffs [52]. *Salmonella* spp. and *Listeria monocytogenes* were not detected in 25 g of the samples, while *Escherichia coli* was present at levels below 10 CFU per gram of sample.

### 3.3. Physicochemical Characterization of Patties: pH, Colour, and Weight Loss

In Table 3, we can see variations in pH, cooking-related losses, and the colour parameters across the different protein matrices (meat, hybrid and plant-based), with and without being subjected to the different treatments: HHP, raw, VC, and a combination of HHP and VC. Table 3 reveals that the treatments significantly affected the pH, weight loss, and colour parameters in the meat, hybrid, and plant-based samples. The hybrid patties showed the greatest pH increase, particularly under combined high hydrostatic pressure and sous vide treatment, while the plant-based samples exhibited minimal changes. Weight loss varied by matrix, with the meat samples experiencing the highest loss under HHP + VC, the hybrid samples showing consistent losses, and the plant-based samples retaining the most moisture. The colour changes were matrix-dependent; the plant-based samples were the most affected, with increased brightness and yellow tones, while the hybrid samples showed complex responses to the treatments.

The treatments exhibited a significant effect on the pH of the samples analysed. No interaction was detected between the treatment and the protein matrix. In the raw samples, the meat had the lowest pH, followed by the hybrid, and finally plant-based patties. After undergoing any treatment, the hybrid samples exhibited an increase in pH values in a higher proportion than that of the plant-based samples. A similar behaviour of the protein matrix was previously reported by Janardhanan et al. in the pH levels of meat, soy-based, and hybrid patties [28].

In all the protein matrices, the combined high hydrostatic pressure and sous vide treatment significantly increased the pH, especially in the meat and hybrid samples (0.7 point increase). The pH changes observed across the different treatment applications may be attributed to a reduction in available acid groups, likely resulting from alterations in protein conformations under applied pressure or temperature [53]. Conversely, in the plant-based samples, the pH remained relatively stable across the HHP and VC treatments, with only the combined treatment (HHP + VC) producing a significant increase of 0.36 points. These findings suggest that the HHP + VC treatment has a more pronounced effect on pH in the hybrid and meat matrices than that in the plant-based matrices. This may be attributed to both the higher protein concentration found in the animal-based protein matrix and the differences in composition (different profiles in acid and basic amino acids) and structure of the plant-based protein matrices [54].

Weight loss data analysis revealed significant variations based on the protein matrix and treatment applied (Table 3), in addition to a significant interaction between the treatment and the protein matrix. For the hybrid samples, no significant difference in weight loss, whether due to exudation or cooking losses, was found when compared to the raw samples, regardless of the treatment employed. Consequently, it is considered that all the hybrid prototypes exhibited weight losses around 6.72 ± 2.00 (referencing the raw state). The variability in the data is attributed to the technological properties of the prototypes, such as the stickiness of the pea flour mixture and fat emulsion as well as the challenges associated with separating the samples from the plastics used for storage before, during, and after the respective treatments. For the meat samples, the combined treatment of HHP and VC led to the greatest weight loss (11.30 ± 5.08) of all the prototypes analysed; these data could suggest an additive/synergistic effect of these treatments on reducing moisture retention. However, considering the high standard deviation of 5.06 (almost as high as the weight loss of the raw sample) and the non-significant changes in the moisture content of the mentioned samples, it can be said that the data do not align with the estimated proximate analysis results. The estimation of cooking-related loss is based on us weighing the samples precooking, and then weighing them after being cooked at a specified internal temperature. Different methods can be used, based on how the samples are handled post-cooking before reweighing: samples can be cooled at room temperature for specifically 5 min, left the samples until they reach ambient temperature, or used ice water immersion to rapidly cool the cooked samples [55]. These differences may have affected the cook-related loss measurements. A potential recommendation includes using a fixed a cooling time for all samples and batches to reduce measurement variability.

In contrast, Janardhanan et al. [28] did not encounter these challenges in effectively measuring weight loss in the samples. Given that the only differences in the composition of the meat patties were increased fat emulsification and the addition of a small amount of xanthan gum, it appears that these modifications were critical to the analysis of this parameter in the meat-based samples. Finally, the plant-based samples exhibited relatively lower weight loss across all the treatments, with the VC treatment showing the least weight loss (1.01 ± 0.71). The combined treatment HHP + VC on the plant-based samples resulted in a weight loss of 1.85 ± 0.79, indicating that the cooking treatments caused less weight loss with respect to the raw samples or those treated only with HHP. The plant-based patties had higher contents of starch and fibre compared to those of the hybrid samples, which are not present in the meat patties. These nutrients could form a network that retains water by hydration and osmotic interactions. Other authors have reported that plant-based products are able to hold fluids better than meat ones [56].

Considering the colour parameters, they were significantly influenced by both the protein matrix and the treatment applied, with a significant interaction between these factors (Table 3). The raw meat samples exhibited less luminosity (L coordinate) compared to those of the plant-based and hybrid samples, with the combination of animal and plant proteins resulting in the brightest appearance, regardless of the treatment applied. L was significantly increased by all the treatments, except for the plant-based samples treated with high pressure, where no significant change was observed.

The values of a* coordinate resembled what was expected; the plant-based samples exhibited green tones, the meat samples showed red tones, and the hybrid samples occupied an intermediate point, with a significant tendency toward green, except in the case of the sous vide-treated samples, which shifted toward redder tones. The treatments had similar effects on this parameter in both the plant-based and meat patties, significantly reducing the green and red tones, respectively, with the effect being more pronounced in the meat patties. The combination of treatments appears to be the main source of colour loss. In the hybrid samples, this parameter exhibited a complex response: high pressure enhanced the green tones. This is consistent with the literature, which indicates that the high-pressure treatment of meat tends to reduce red tones due to the oxidation of ferrous myoglobin to ferric myoglobin [20,57]. Meanwhile, the sous vide treatment enhanced the red tones, potentially due to the thermal degradation of chlorophyll, a vegetal pigment present in pea flour responsible for the green colouration in most plant-based products [58]. However, the combination of treatments did not significantly affect the sample colour, suggesting that these effects may have counterbalanced each other. The b* coordinate tended toward positive values in all the samples, regardless of the matrix, indicating a trend toward a yellow hue most notably in the plant-based samples, followed by the hybrid and meat samples. In the plant-based samples, yellow tones increased across all the treatments, with the following order of impact: HHP + sous vide > sous vide > HHP. For the hybrid samples, only sous vide (either alone or combined with HHP) significantly increased the yellow tones. In the meat samples, sous vide cooking (alone or in combination) produced a noticeable increase in this coordinate. In contrast to the findings with soy-based samples by Janardhanan et al. [28], pea flour, an allergen-free plant-based protein matrix, is highly susceptible to colour modifications when treated with HHP or sous vide.

Colour in pea flour is dependent on the protein it contains [59], and it is known that HHP and VC treatments cause the denaturation and modification of the properties of food proteins [60]. The modifications in the present study have resulted in either a reduction in green tones or an increase in yellow tones and luminosity. This susceptibility may present both an advantage and a limitation, depending on how this raw material is utilised to replace its main competitor, soy, in plant-based protein formulations.

### 3.4. Instrumental Texture of Patties

The results of texture analysis are shown in Table 4 for each protein matrix. The data demonstrate significant differences in the texture attributes depending on the applied treatments. The plant-based samples consistently showed the highest hardness and gumminess, particularly after the combined high-pressure and sous vide (HHP + VC) treatment, which tripled the degree of gumminess compared to that of the raw ones. The meat samples were the least affected by the treatments, with stable hardness and lower values for hardness, gumminess, and chewiness. The hybrid samples exhibited intermediate texture properties, with notable increases in hardness and gumminess under certain treatments, suggesting synergistic effects between the meat and pea proteins for these attributes.

The plant based patties exhibited the highest hardness at 1.08 N, followed by the hybrid samples at 0.34 N and the meat samples at 0.27 N. Across all the treatments, the plant-based samples consistently demonstrated the highest hardness, showing a significant increase in this attribute when submitted to any treatment. The maximum peak of hardness was observed for each protein matrix when subjected to the combined treatment of high pressure and sous vide, reaching 4.24 N (maximum value) in the plant-based samples, doubling the value achieved by the treatments applied individually. The hardness hierarchy remained consistent across the treatments: plant-based > hybrid > meat. The greatest impact on firmness was observed in the hybrid and plant-based patties. In the raw and high-pressure-treated samples, the hybrid and meat patties exhibited similar hardness levels. However, sous vide cooking, with or without high-pressure treatment, significantly increased the hardness of the hybrid samples, though less so compared to the plant-based samples. It is noteworthy that high-pressure treatment affects non-covalent bonds and can alter biopolymer structures, including protein denaturation [61,62]. As a result, denatured proteins can experience a reduction in their water-holding capacity [63]. Since the meat and hybrid samples have higher water and protein contents, this reduction in water-holding capacity may diminish the emulsifying capacity of the hydrogel used, resulting in a less-firm texture in the samples treated solely with high pressure. Comparing the results from this study to those reported by Janardhanan et al. [19], clear differences emerge. In the earlier study, the values for hardness were significantly higher than those in the present research; however, both the studies observed similar trends, with the samples subjected to treatment combination (HHP + sous vide) and the plant-based matrices consistently showing the greatest hardness. The previous study also reported higher standard deviations, indicating greater heterogeneity within the samples compared to that in this work. These differences may stem from variations in sample composition (e.g., higher content of fat emulsion) and differences in the plant-based protein matrices used [64]. Finally, focusing on the protein matrix, the results were compared to those of Afshari et al. [65], which found a 13% higher increase in hardness in plant-based prototypes where meat fat was replaced with a canola and olive oil blend relative to that of the control samples containing meat. These findings align with the present study, where the plant-based samples exhibited higher hardness levels than those of the meat samples. The lower moisture content of the plant-based patties may influence the higher values observed and on the greater impact of the technologies applied.

As shown in Table 4, the springiness behaviour of the samples contrasts with their hardness. For all the three matrices, sous vide treatment (whether combined with other methods or not) tended to decrease this parameter, while high hydrostatic pressure alone did not affect their springiness. The plant-based samples consistently exhibited the lowest springiness values across all the treatment categories. The present data contrast with the previous findings by Janardhanan et al. [20], who observed that springiness in certain meat samples was maintained or even improved following high-hydrostatic-pressure treatments. Additionally, they reported that plant-based food exhibited lower springiness values than those of the meat samples [28], suggesting a comparable springiness behaviour between pea-based and soy-based formulations.

In the case of cohesiveness, the plant-based samples exhibited lower values than the meat and hybrid samples, similar to the trend observed with springiness. This is logical as higher values for both the parameters indicate a lower tendency to deform, and according to Petracci and Cavani [66], the presence of intramuscular connective tissue, a natural component of meat, may be one of the primary factors contributing to the high cohesiveness observed in meat-based food products. High hydrostatic pressure did not significantly increase the cohesiveness of any sample; however, a reduction in this parameter was observed in those samples subjected to the sous vide treatment (whether combined or not).

The plant-based samples exhibited higher gumminess values compared to those of both the meat and hybrid samples, regardless of the treatment applied. The combination of treatments significantly enhanced the gumminess of both the hybrid and plant-based samples.

With respect to chewiness, the raw samples showed similar chewiness values across all the protein matrices. Notably, chewiness remained constant in the hybrid samples across all the treatments, while in the plant-based patties, a significant increase in chewiness was observed with the combined HHP+VC treatment. The meat samples appeared to be negatively affected by all the treatments, with chewiness reduced in every case. Previous studies on meat analogues made from beef and pork have successfully achieved comparable hardness to the original products, minimizing the significant differences in texture [67]. However, replicating the characteristic hardness and springiness of chicken meat proves to be more challenging, given the unique textural complexity of chicken compared to that of other meat types [68]. Springiness is generally one of the most challenging textural attributes to replicate in meat analogues, reducing consumer acceptance of this product category [68]. For the present study, the significant differences between meat analogues and traditional meat prototypes is not desirable, as it hinders consumer acceptance of these new plant-based products. However, the presence of hybrid products with intermediate values has been positively highlighted, as they seem to retain the textural advantages of meat (to which consumers are already accustomed [69]), while incorporating the beneficial attributes of plant-based products. Regarding the data obtained by Janardhanan et al. (2023) [18], their findings show higher values for most of the parameters analysed (both meat and soy-based samples). A review of the experimental process suggests that while the addition of xanthan gum enhanced emulsion stability, it may have reduced firmness, resulting in less-solid prototypes. The literature confirms that xanthan gum can influence the polymerisation of other gums (e.g., κ-carrageenan), either positively or negatively, depending on the food matrix used [70,71,72]. Based on the results obtained, despite the increase in emulsion stability it provides, we do not recommend formulating with this gum in its composition, as it has been shown to significantly reduce the firmness of samples.

### 3.5. Sensory Analysis of Patties

Following visual characterization, the following figures provides a visual representation of the data collected from the 16 panellists across the various variables for the three different prototypes using radar charts.

**Figure 3:**
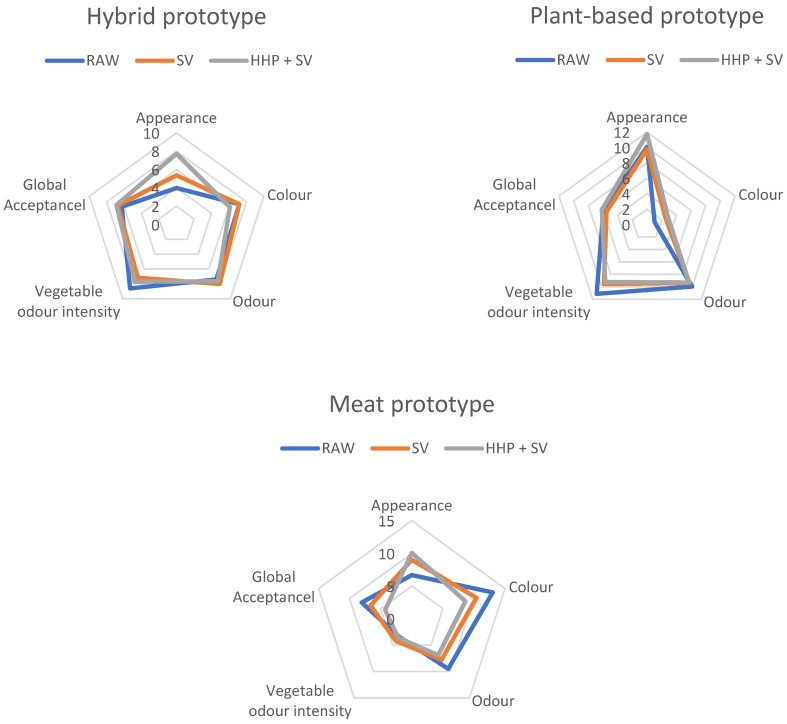
Sensory profiles of samples raw and treated by vacuum cooking and combination of high hydrostatic pressure + vacuum cooking according to different protein matrices: meat-based, plant-based, and hybrid patties. RAW: raw samples; SV: samples treated sous vide; HHP + SV: samples subjected to sous vide and high hydrostatic pressure.

In the meat samples, as illustrated in Figure 3, pinkish and brownish tones are predominant in the raw state. These colours become less intense following either sous vide or HHP treatment, indicating a loss in colour vibrancy due to cooking. This reduction in colour intensity aligns with the colorimetric data, suggesting that treatment application affects the dispersion and consistency of colour, particularly in meat samples, where a decrease in red tones is notable. Figure 3 also highlights a trend towards increased visual homogeneity in the meat matrices after any treatment, indicating that both sous vide and HHP stabilise the appearance of these samples. Regarding odour analysis, the panellists identified an unpleasant and less-appetizing scent profile in the meat samples, lacking the fresh odour quality observed in the plant-based and hybrid matrices. Furthermore, the meat samples display the lowest intensity of vegetable odour among the three matrices, with minimal influence from the treatments. In terms of global acceptance, subtle variations indicate that the raw samples receive slightly higher acceptance than the treated samples, especially the HHP-treated ones. This suggests that the increase in homogeneity, the emergence of more rancid odours, and the loss of brown and pink tones were not well received by the panellists in the trial.

The plant-based samples are concentrated at the green end of the colour spectrum in their raw form. Similar to the meat samples, the plant-based samples experience a loss of colour intensity after applying either the sous vide or HHP treatment, though green tones remain dominant. Colorimetric analysis supports these findings, indicating a decrease in red and green hue intensity across all the treatments. In terms of visual homogeneity, the plant-based matrices are perceived as highly consistent and homogeneous, maintaining this appearance even without treatment, and indicating a stable visual quality. Odour analysis indicates the slight predominance of a fresh odour in the plant-based samples. Given the complexity of odour characterization, the panellists were invited to describe the scents that most captured their attention. Notably, the samples containing pea flour exhibited prevalent scents of legumes, animal feed, grass, and cereals. Figure 3 further highlights that plant-based samples exhibit the highest intensity of vegetable odour across all the matrices, an attribute that remains largely unaffected by either treatment, though the raw plant-based samples tended to have a slightly stronger vegetable scent than that of the treated ones. The overall acceptability of the samples exhibited considerable variability among the panellists. Similar to the other samples, the ratings generally were around a neutral midpoint between like and dislike, regardless of treatment application.

Figure 3 shows that the colour distribution of the hybrid sample in its raw state presents a blend of green and brownish/pinkish tones, appearing as an intermediate point between the meat and plant-based samples. This colour combination remains relatively stable with the application of either sous vide cooking or high-pressure processing combined with sous vide. According to the colorimetric evaluations, these treatments minimally affect the dispersion and consistency of colour in the hybrid samples, though a general reduction in the predominant red and green hues was noted. Figure 3 further reveals that the hybrid matrices displayed a mostly heterogeneous appearance, which became slightly more homogeneous when subjected to HHP. Moreover, colour analysis indicates that the HHP-treated hybrid samples exhibited lower standard deviations in colour compared to those of the untreated samples, aligning with the panellists’ visual assessments. This outcome is consistent with the expected effect of high-pressure treatment on foods, which tends to standardise sensory properties such as colour [73]. In terms of odour, the panellists reported a lack of consensus on the dominant odour of the hybrid samples, detecting a mixture of scents similar to those in the plant-based samples. The hybrid samples also demonstrate an intermediate level of vegetable odour intensity, with the raw samples showing a slightly higher intensity. However, the treatments appear to have no significant effect on this parameter.

The heterogeneity of sensory evaluation responses can be attributed to individual differences in sensory sensitivity and perception. However, in this case, there may be additional factors at play. The panellists could be influenced by the product’s name (e.g., “plant-based” or “hybrid”), as expectations surrounding such products can vary widely, which, in turn, affect how non-hedonic attributes are perceived [74]. Moreover, unfamiliarity with the hybrid textures or flavour combinations in the intermediate hybrid samples may result in varying levels of acceptance [75]. These factors, along with individual preferences and sensory thresholds, contribute to the observed variability in sensory analysis.

### 3.6. Carbon Footprint of Patties

Table 5 presents the detailed analysis of the carbon footprints associated with the three types of patty prototype (meat, plant-based, and hybrid). The meat-based prototype amounts to 1.62 kg CO_2_e per patty (150 g), the hybrid prototype is associated with 0.87 kg CO_2_e, while the plant-based one is associated with 0.12 kg CO_2_e.

In addition to the protein matrices, all the three patties contain several common ingredients, such as broccoli extract (to enhance nutritional quality), salt, olive oil, linseed oil, water, kappa-carrageenan, xanthan gum, and polysorbate. Among these, broccoli extract has the highest emission factor of about 9.91 kg CO_2_ eq/kg. As already mentioned, agro-industrial by-products currently have no economic value (economic factor 0); therefore, the total impact of the cultivation phase is attributed to broccoli production. Thus, the broccoli extract used for the prototypes is not associated with greenhouse gas emissions from the broccoli production phase [17]. The reported emissions result mainly from industrial processes used for glucosinolate extraction. In fact, in recent years, extraction processes have been identified as an important source of greenhouse gases due to the substantial use of chemical solvents. Attempts are being implemented based on emerging technologies to find more sustainable methods for bioactive extraction from plant material [79]. The other ingredients (salt, water, and emulsifiers) contribute minimally to the overall footprint of the meat and hybrid patties, whereas in the plant-based patty, they emit more emissions than the protein matrix.

During the preparation of the patties, the primary potential source of greenhouse gas emissions is the electricity consumption required for processing activities. However, since the pilot plant facilities were powered exclusively by a renewable energy provider, no greenhouse gas emissions were generated in this phase of production [46]. The renewable energy source effectively eliminates the carbon footprint associated with electricity use in patty preparation, thereby contributing to a lower overall environmental impact for this stage of the production life cycle [47].

The meat patty, primarily composed of meat (72.63%), has the largest carbon footprint of the three types, with an emission total of 1.62 kg CO_2_ eq per patty. This significant footprint is due to the high emission factor for meat, estimated at 13.93 kg CO_2_ eq per kilogram for meat with a protected designation of origin produced in Navarra, Spain. The largest source of CO_2_ emissions in the meat production system is the feed cultivation and preparation phase, amounting to 91% of the total greenhouse gas emissions [47]. Meat’s larger carbon footprint is consistent with other research [80], reflecting the resources needed in animal agriculture, such as feed, water, land, and wastage management. In contrast, the plant-based patties, which consist of 72.63% hydrated green pea flour as the protein matrix, has the smallest carbon footprint, at just 0.12 kg CO_2_ eq per patty. This low emission total is due to the much lower emission factor for hydrated green pea flour, calculated at 0.13 kg CO_2_ eq per kilogram. The data for green pea flour are calculated from production in the region of Navarra, Spain (the results can be seen in the Supplementary Material, Table S1: activities and emissions of the green pea cultivation carbon footprint, scopes 1 and 2 (2018)). This substantial difference in emissions is due to the fact that production requires fewer resources and results in lower greenhouse gas emission totals than those of the animal-based products [81]. In this case, the carbon impact generated by flour is so low that the relative importance of the other ingredients increases, with broccoli extract emerging as the primary source of impact in these samples. The hybrid patty combines meat and hydrated green pea flour, each constituting 36.31% of its composition. This blend results in a carbon footprint total of 0.87 kg CO_2_ eq per patty, reducing the emissions by nearly half compared to that of the meat patties, while maintaining its nutritional benefits [82].

Table 6 compares the environmental impacts of the meat and plant-based patties from different countries. In line with the previous studies, meat products are shown to have a much larger carbon footprint compared to that of plant-based alternatives [83]. It is important to note that none of the reviewed studies, including the present one, accounted for the environmental impact of packaging, ensuring the comparability of the results across studies. However, the carbon footprints recorded in this study are substantially smaller than those reported in the literature (see Table 6). This reduction is primarily due to the use of PGI-certified meat, which relies on more sustainable resource management, leading to fewer greenhouse gas emissions for meat-based products. For plant-based samples, prior studies often include products with a broader diversity of ingredients and highly processed legumes, which contribute to higher associated emissions. This also highlights the variability in environmental impact due to differences in processing and ingredient sourcing. Life cycle assessment analyses underscore that the environmental footprint of food products is strongly influenced by the origin and production methods of the ingredients used. The key factors, such as the local climate, the geographical location, deforestation for crop cultivation (e.g., soy), and energy-intensive processing methods, significantly impact the final results [84].

In Heller and Keoleian [85]’s study case, it was reported that the greenhouse gas emission totals of plant-based options represent approximately 10.8% of those associated with beef burgers. Additionally, Takacs et al. [89] compared the carbon footprints of 14 dishes with different protein matrices, divided between vegan and vegetarian meat analogues and meat dishes, and concluded that vegan food products generally produce about 7.1% of the greenhouse gas emissions compared to their meat-based counterparts. Similarly, Dominguez-LaCueva et al. [47] found that soy-based patties emitted only 7.6% of the greenhouse gases that beef patties do, while the present study underscores confirm this trend with pea-based patties (7.3%).

This evaluation aligns with the recent literature and underscores the environmental differences between patty types based on their protein composition. The high emission totals associated with the meat patty highlight the environmental costs of animal-based products, while the plant-based patty emerges as the most sustainable option. The hybrid patty presents a balanced compromise, effectively reducing the carbon footprint of food systems by combining meat with plant-based ingredients, while preserving nutritional quality. This approach supports the notion that the complete elimination of animal production systems is unnecessary, advocating instead for improved management and reduced dependency on such systems [90]. This discussion reinforces the value of adopting more hybrid options within the Navarrese food chain and contributes to carbon emission reductions in line with the Paris Agreement [91].

## 4. Conclusions

This study provides valuable insights into the development of alternative protein products that address both the dietary and environmental challenges associated with traditional meat consumption. The reduced carbon footprint of the plant-based patties highlights their potential as a highly sustainable option, particularly when contrasted with the significant emissions linked to the meat patties, which exemplify the environmental cost of animal-based products. The hybrid patties, on the other hand, represent a promising intermediate solution by integrating meat and plant-based components. This combination not only reduces carbon emissions, but also enhances nutritional quality, as the inclusion of both animal and plant-derived ingredients contributes to a more balanced nutritional profile. Furthermore, the findings suggest that the hybrid patties are a promising alternative to our plant-based products, as they offer a higher protein content. This approach supports the gradual shift toward more sustainable protein options within the Navarrese food chain.

The prototypes developed in this study also present notable nutritional potential. While no regulated guidelines currently exist for glucosinolate intake, the antioxidant and anti-inflammatory properties of these compounds, as well as their potential role in modulating carcinogenic processes, add value to the plant-based formulations. Additionally, utilizing by-products for glucosinolate extraction underscores the commitment to sustainability by minimizing resource use in production. From a technical perspective, the choice of native pea flour over extruded soy as the protein base aligns with the goal of reducing allergenicity. However, future research should prioritise the investigation of the impact of native and extruded pea flour on these formulations, with a focus on ensuring the digestibility and the micronutritional quality of the prototypes, while maintaining their allergen-free benefits.

Regarding the treatments, the hybrid samples exhibited an increase in pH due to the combined application of treatments. However, this did not compromise the microbiological safety of the samples, confirming the food safety properties conferred by the treatments. The combination of treatments also enhanced the homogeneity of the hybrid samples and reduced the presence of vegetal odours. Nevertheless, these changes did not appear to influence the hedonic acceptance (visual or olfactory) by the panellists. In terms of texture, hardness and gumminess were improved by the combined application of the treatments. However, not all the effects were positive; losses in springiness and cohesiveness were observed.

Further research is required to explore whether adjustments to the duration and intensity of the processes could preserve the positive textural attributes without compromising the others. Additionally, sensory challenges, such as changes in appearance and odour in the meat-based samples, underscore the need for further refinement. These findings highlight the importance of ongoing efforts to optimise sensory attributes and nutritional value, while balancing sustainability and consumer acceptance.

## Figures and Tables

**Table 1:** Percentage composition by ingredient for meat, hybrid, and plant-based patties (NA: not applicable).

Ingredients (%)	Patties		
	Meat	Hybrid	Plant-Based
Beef meat	72.6	36.3	NA
Pea meal	NA	36.3	72.6
Emulsion	21.3	21.3	21.3
Broccoli extract	4.7	4.7	4.7
Salt	1.4	1.4	1.4

**Table 2:** Proximal composition of meat, plant-based, and hybrid patties (with and without treatments). Values are expressed as mean (standard deviation).

Protein Matrix	Treatment	Moisture%	Ashes%	Protein%	Fat%
Hybrid samples	Raw	53.13 (0.43)	3.72 (0.07) ^a^	15.48 (0.23) ^a^	7.95 (0.15)
	HHP	52.91 (0.42)	2.87 (0.23) ^b^	14.72 (0.45) ^ab^	8.12 (0.31)
	VC	53.08 (0.18)	2.74 (0.12) ^b^	14.27 (0.49) ^b^	8.36 (0.12)
	HHP + VC	52.77 (0.28)	2.91 (0.10) ^b^	14.47 (0.34) ^b^	7.98 (0.28)
Meat samples	Raw	68.44 (0.23) ^a^	2.39 (0.30)	17.56 (0.19) ^ab^	7.09 (0.15) ^a^
	HHP	67.68 (0.51) ^b^	2.63 (0.12)	16.90 (0.41) ^b^	6.99 (0.16) ^a^
	VC	67.70 (0.33) ^b^	2.45 (0.16)	17.69 (0.58) ^ab^	6.11 (0.35) ^b^
	HHP + VC	68.45 (0.60) ^a^	2.57 (0.25)	17.90 (0.60) ^a^	6.51 (0.20) ^ab^
Plant-based samples	Raw	37.86 (0.29)	3.16 (0.07)	11.87 (0.06) ^a^	7.53 (0.02) ^b^
	HHP	37.70 (0.36)	2.78 (0.33)	12.09 (0.06) ^a^	7.98 (0.10) ^a^
	VC	38.59 (1.14)	2.91 (0.25)	11.44 (0.30) ^b^	7.86 (0.19) ^ab^
	HHP + VC	38.12 (0.40)	2.89 (0.07)	11.98 (0.08) ^a^	7.52 (0.13) ^b^

Different superscripts in same column indicate significant differences (*p* < 0.05) using Tukey’s test. Comparisons were made within same protein matrix. Raw: raw samples; HHP: samples treated under high hydrostatic pressure; VC: samples treated sous vide; HHP + VC: samples subjected to both treatments.

**Table 3:** pH, weight loss, and colour parameters of meat, plant-based, and hybrid patties (with and without treatment). Values are expressed as mean (standard deviation).

Protein Matrix	Treatment	pH	Weight Loss%	Luminosity	Coordinate a*	Coordinate b*
Hybrid samples	Raw	5.59 (0.04) ^b^	6.72 (2.00)	43.89 (2.82) ^d^	−0.54 (1.28) ^b^	36.38 (4.27) ^b^
	HHP	5.81 (0.07) ^b^	4.08 (3.54)	47.08 (1.34) ^b^	−1.67 (0.80) ^c^	37.71 (4.13) ^b^
	VC	5.72 (0.09) ^b^	4.62 (1.05)	45.80 (1.40) ^c^	0.35 (0.76) ^a^	43.41 (5.87) ^a^
	HHP + VC	6.29 (0.48) ^a^	5.41 (1.54)	48.83 (0.70) ^a^	−0.84 (0.71) ^b^	45.11 (1.16) ^a^
Meat samples	Raw	5.29 (0.03) ^c^	5.77 (1.61) ^b^	32.06 (2.80) ^d^	16.79 (1.66) ^a^	27.33 (3.08) ^b^
	HHP	5.55 (0.04) ^b^	7.55 (2.64) ^ab^	44.17 (1.71) ^b^	10.50 (1.50) ^c^	27.11 (4.30) ^b^
	VC	5.49 (0.05) ^bc^	3.42 (0.46) ^b^	40.83 (2.15) ^c^	13.42 (1.70) ^b^	30.45 (3.67) ^a^
	HHP + VC	5.97 (0.47) ^a^	11.30 (5.08) ^a^	46.41 (2.71) ^a^	7.98 (1.33) ^d^	29.39 (2.62) ^ab^
Plant-based samples	Raw	5.75 (0.03) ^b^	5.65 (2.63) ^a^	38.19 (1.24) ^b^	−10.68 (0.51) ^c^	49.87 (5.59) ^c^
	HHP	5.75 (0.05) ^b^	6.43 (6.22) ^a^	37.82 (1.23) ^b^	−9.25 (1.19) ^b^	53.81 (7.80) ^c^
	VC	5.71 (0.03) ^b^	1.01 (0.71) ^b^	41.01 (2.50) ^a^	−7.35 (1.66) ^a^	57.59 (6.45) ^ab^
	HHP + VC	6.11 (0.46) ^a^	1.85 (0.79) ^b^	40.82 (0.54) ^a^	−7.25 (0.39) ^a^	61.98 (1.60) ^a^

Different superscripts in same column indicate significant differences (*p* < 0.05) using Tukey’s test. Comparisons were made within same protein matrix. Raw: raw samples; HHP: samples treated under high hydrostatic pressure; VC: samples treated sous vide; HHP + VC: samples subjected to both treatments.

**Table 4:** The texture parameters of the samples when raw and subjected to the different treatments. The values are expressed as means (standard deviation).

Protein Matrix	Treatment	Hardness (N)	Springiness	Cohesiveness	Gumminess	Chewiness (N)
Hybrid samples	Raw	0.35 (0.10) ^b^	0.77 (0.26) ^a^	0.46 (0.18) ^a^	0.16 (0.09) ^b^	0.13 (0.08)
	HHP	0.33 (0.11) ^b^	0.86 (0.13) ^a^	0.50 (0.09) ^a^	0.18 (0.08) ^b^	0.15 (0.07)
	VC	1.13 (0.76) ^a^	0.44 (0.46) ^b^	0.29 (0.10) ^b^	0.28 (0.18) ^b^	0.24 (0.34)
	HHP + VC	1.48 (1.09) ^a^	0.40 (0.12) ^b^	0.31 (0.06) ^b^	0.45 (0.33) ^a^	0.18 (0.16)
Meat samples	Raw	0.28 (0.05) ^ab^	0.78 (0.16) ^a^	0.44 (0.10) ^a^	0.12 (0.04) ^a^	0.10 (0.05) ^a^
	HHP	0.21 (0.07) ^b^	0.74 (0.18) ^a^	0.41 (0.10) ^b^	0.08 (0.03) ^bc^	0.07 (0.04) ^b^
	VC	0.22 (0.05) ^ab^	0.47 (0.19) ^b^	0.31 (0.07) ^c^	0.07 (0.01) ^c^	0.03 (0.04) ^c^
	HHP + VC	0.30 (0.25) ^a^	0.55 (0.23) ^b^	0.36 (0.08) ^b^	0.10 (0.08) ^ab^	0.05 (0.03) ^bc^
Plant-based samples	Raw	1.08 (0.52) ^c^	0.43 (0.63) ^a^	0.25 (0.13) ^a^	0.25 (0.17) ^b^	0.09 (0.14) ^b^
	HHP	2.65 (2.83) ^b^	0.33 (0.21) ^ab^	0.25 (0.10) ^a^	0.55 (0.69) ^b^	0.10 (0.06) ^b^
	VC	2.07 (1.24) ^bc^	0.16 (0.8) ^b^	0.14 (0.07) ^b^	0.32 (0.30) ^b^	0.05 (0.06) ^b^
	HHP + VC	4.24 (2.21) ^a^	0.24 (0.07) ^ab^	0.19 (0.05) ^ab^	0.88 (0.54) ^a^	0.22 (0.17) ^a^

The different superscripts in the same column indicate significant differences (*p* < 0.05) using Tukey’s test. Comparisons were made within the same protein matrix. Raw: raw samples; HHP: samples treated under high hydrostatic pressure; VC: samples treated by sous vide; HHP+VC: samples subjected to both the treatments.

**Table 5:** The data, calculations, and literature sources of the carbon footprints of the developed prototypes.

Prototype	Ingredients	%	Quantity/Patty (150 g)	Emission Factor kg CO_2_ eq/kg	Reference	Origin	kg CO_2_ eq/1 ud of Meat Patty	kg CO_2_ eq/1 ud of Hybrid Patty	kg CO_2_ eq/1 ud of Plant-Based Patty
Meat patty	Meat	72.63	108.94	13.93	[47]	Navarra	1.52	-	-
Plant-based patty	Green pea flour	72.63	108.94	0.13	Present study	Navarra	-	-	0.014
Hybrid patty	Meat	36.31	54.47	13.93	[47]	Navarra	-	0.76	-
	Green pea flour	36.31	54.47	0.13	Present study	Navarra	-	0.007	-
Common ingredients	Broccoli extract	4.69	7.03	9.91	[17]	Navarra	0.07	0.07	0.07
	Salt	1.41	2.11	0.60	[76]	France	0.001	0.001	0.001
	Olive oil	5.07	7.60	2.22	[77]	South Spain	0.02	0.02	0.02
	Linseed oil	3.38	5.07	3.54	[76]	France	0.02	0.02	0.02
	Water	12.35	18.52	1.35 × 10^−3^	[78]	Italy	2.49 × 10^−5^	2.49 × 10^−5^	2.49 × 10^−5^
	Kappa-carrageenan	0.32	0.48	-		-	-	-	-
	Xanthan gum	0.16	0.24	-		-	-	-	-
	Polysorbate	0.01	0.02	-		-	-	-	-
Processing	Meat			0	[46,47]	UPNA Navarra	0	0	0
	Plant-based			0					
	Hybrid			0					
Total result							1.62	0.87	0.12

**Table 6:** Carbon footprints of meat, plant-based, and hybrid patty production in consulted literature (without considering packaging).

Meat Patty (150 g)	Hybrid Patty (150 g)	Plant-Based Patty (150 g)	Source	Note	Plant-Protein Ingredient
1.62	0.87	0.12	Present study	Navarra, Spain	Native Pea
2.09	1.09	0.16	[47]	Navarra, Spain	Extruded Soy
4.81	-	0.53	[85] ^1^	North America	Isolated Pea
4.67	-	0.2	[86] ^1^	Sweden	Isolated Pea
8.76			[87] ^2^	Ireland Brazil United Kingdom	Mix of legumes
	5.97				
		1.99			
-	0.41	0.32	[88] ^2,3^	Global	Average of Soy-based products

^1^ The carbon footprint includes transportation. ^2^ The carbon footprint includes transportation and packaging emissions. ^3^ Meat analogues are made with egg.

## Data Availability

The original contributions presented in this study are included in the article/Supplementary Material. Further inquiries can be directed to the corresponding author.

## Supplementary Materials

The following supporting information can be downloaded at: https://www.mdpi.com/article/10.3390/foods14030472/s1, Survey S1. Survey distributed to farmers (translated to English). Table S1. Activities and emissions of the green pea cultivation carbon footprint, Scope 1 and 2 (2018). Text S1. Data on plot and farming systems. Refs. [92,93] are cited in Supplementary Materials.

## Abbreviations

HHP: high hydrostatic pressure; UPNA: Public University of Navarra; VC: vacuum cooking (sous vide cooking); TPA: texture profile analysis; CO_2_e: CO_2_ equivalents; PGI: Protected Geographical Indication.
