# Supplementary material for: Plant-Based and Hybrid Patties with Healthy Fats and Broccoli Extract Fortification: More Balanced, Environmentally Friendly Alternative to Meat Prototypes?
Source: Foods. 2025 Feb 1;14(3):472. doi: 10.3390/foods14030472 (PMC11816437; doi:10.3390/foods14030472)
Supplement: Supplementary file 1 [file foods-14-00472-s001.zip › foods-3359337-supplementary.pdf]

Supplementary Material

Survey S1/ Survey distributed to farmers (translated to English).

The ALISSEC project aims to generate new food products. Within the framework of this project, the aim is to carry out a sustainability analysis of the resulting products, and for this, we need your help:

This questionnaire collects data on the crops used, if it is not possible to obtain direct measurements of what is asked, estimates are welcome (please clarify when data are estimates).

General data: Green Pea

1. **Crop flow diagram**

The following steps will be taken into account for the study, if there is any other operation that you think needs to be taken into account in the cultivation process, please let us know below.

PRE-APPLANTED PLOWING → SOWING/TRANSPLANTING → TREATMENTS AND FERTILISATION → HARVESTING

2. **During pre-ploughing, what type of implement is used and how many passes are made over the field on average?**
   Two chisel passes
   One cultivator pass

3. **Places of cultivation and cultivated area:** *(names, coordinates or nearby points is sufficient and hectares)*
   Navarra, Cirauqui, polygon 9, plot 457area 1
   Surface area: 2 hectares

4. **Sowing date**: 29/11/2020

5. **Harvest date:** 04/07/2021

6. **Harvest quantities** *(e.g. total kg produced and/or kilograms produced per hectare):*
   7020 kg / ha.

7. **What irrigation system is used? And do you have direct measurements of daily irrigation for the crop?** *(e.g. drip irrigation, 250 m3/day*ha) If yes, please provide it:*
   NA

   If no, can you describe how the farmer decides when to irrigate and give an approximate indication of the amount of water applied in total, or per unit area?
   NA

8. **If water is used directly for some process other than irrigation, what process is that and approximately how much water is used?** *(e.g.: frost protection, pesticide distribution, crop clean-up…)*
   It is not used

9. **Which water basin(s) do you depend on for the water supplied to your activities?**
   NA

10. **Are fertilisers, pesticides, manure, compost, sludge or limestone applied? Which ones? How much** (*kg/ha*)?:
    Fertilizer: (9-23-30) 1418 Kg. 700 Kg/ha

11. **After harvesting, are crop residues burnt, collected or left on the ground? Is there an estimated quantity?** *Indicate whether the remains are chopped and/or incorporated by turning.*
Crop residues are left on the ground, then incorporated into the soil.

12. **Are fuels consumed in <u>fixed installations</u> related to this crop?** If yes **What type of fuel and quantity consumed?**, *Please complete the table (Boilers, water pumps, heating, cooling, turbines, furnaces, ovens),*

Gasoline, Diesel A, Natural gas, Butane, Propane, Biomass, Domestic or imported coal, Other (specify):

Installation	Fuel Type	Relative quantity for this crop (total, daily, weekly, monthly)
NA	NA	NA

13. **If there is fuel consumption in tillage or crop management, type of fuel and distance travelled?** *Please specify what it is due to (tractors, harvesters, portable irrigation machines, truck) (if exact information is not known, where possible, provide km/hectares travelled, type of transport and type of fuel).*

Petrol, diesel A, diesel B, KWh (electric vehicles), E(5), B(7), other (specify)**:**

Vehicle	Fuel	Km travelled	Relative quantity for this crop (total, daily, weekly, monthly)	Reason
Tractor (Jonh Deere)	Diesel	25 minutes	Total	Field operations

14. **If there is consumption of fluorinated gases in the period described, type of fluorinated gas and quantity consumed?** *(recharged or leaking) (e.g. refrigeration and air conditioning, fire extinguishers, Aerosols…)*
NA

15. **Which energy supplier is contracted, how many kWh were consumed in the months of cultivation?** *(Iberdrola, Iberdrola clientes, CIE Energía S.L., etc.) (Indicate if they have any guarantee of origin: renewable energy or high efficiency cogeneration and electricity consumption).*
Not applicable

16. **Is there any source of energy production on the farm? If yes, how many KWh were produced in the growing period?**
Not applicable

17. **Which of the following land management systems most closely resembles the one used on your farm.**

☒ **Traditional farming:** *(removing/disturbing by mechanical methods the soil to a depth of 20 cm or more)*

☐ **Minimal tillage:** *(surface tillage by cultivators, harrows or ploughs, at a depth of less than 20 cm.)*

☐ **Spontaneous vegetation cover:** *(No mechanical tillage, with spontaneous vegetation, whose growth is controlled mechanically, chemically or by grazing.)*

☐ **Vegetation cover sown:** *(No mechanical tillage, soil protected by vegetation cover sown with grasses or legumes, the growth of which is controlled mechanically, chemically or by grazing.)*

☐ **Inert covers:** *(soil covered with pruning waste, stones or inert compounds.)*

☐ **No maintenance:** *(No maintenance in the last campaign)*

☐ **No tillage:** *(No mechanical tillage, no plant cultivation and compaction problems appear).*

18. **Are single-use materials used, and if so, do you know the approximate quantities?** *E.g. raffia bags, mulching plastics, crop protection, others (for plastics, please clarify the type of plastic used: PET, PP, HDPE, LDPE, PVC…).*
No

Table S1/ Activities and emissions of the green pea cultivation carbon footprint, Scope 1 and 2 (2018).

		kg CO_2	g CH_4	g N_2O	kg CO_2e
**DIRECT EMISSIONS* (SCOPE 1)**	**Fertilizers, amendments, burning and management of agricultural residues.**	0.00	0.00	8,091.13	2,144.15
	**Fixed installations**	0.00	0.00	0.00	0.00
	**Road transport**	0.00	0.00	0.00	0.00
	**Operation of machinery**	247.11	2.30	10.86	250.05
	**Rail, maritime and air transport**	0.00	0.00	0.00	0.00
	**Fugitive emissions - air conditioning and refrigeration**	0.00	0.00	0.00	0.00
	**Process**	0.00	0.00	0.00	0.00
	**SUB-TOTAL**	**247.11**	**2.30**	**8,101.99**	**2,394.20**
**INDIRECT EMISSIONS* ELECTRICITY AND OTHER ENERGIES (SCOPE 2)**	**Electricity in buildings**	-	-	-	0.00
	**Electricity for vehicles**	-	-	-	0.00
	**Heat, steam, cold, compressed air**	0.00	0.00	0.00	0.00
	**SUB-TOTAL**	**0.00**	**0.00**	**0.00**	**0.00**
**TOTAL**		**247.11**	**2.30**	**8,101.99**	**2,394.20**

*[46] MITECO, Huella de carbono de una explotación agrícola. Alcance 1+2. (2022). Madrid, Spain. Accessed: Aug. 20, 2023. [Online]. Available: https://www.miteco.gob.es/content/dam/miteco/es/cambio-climatico/temas/mitigacion-politicas-y-medidas/calculadora_hc_agri_tcm30-485620.xlsx

Text S1/ Data on plot and farming systems

The cultivation plot was located in the south-western region of Navarre (Spain), specifically in the cultivable area of Cirauqui. The surveys collected detailed information on various aspects of the farming process, including crop stages and timelines, the type of crops harvested, the total hectares cultivated, the quantity and type of fertilisers used, the equipment and machinery employed, crop residue management practices, and the tillage methods applied. Emission factors for 2021 and calculation protocol were sourced from the MITECO calculator [46]. Agricultural work was carried out using a tractor with two different implements, including two chisel plow passes and one cultivator pass. Harvesting of the green peas was performed manually. Regarding the refining and flour production phase of the peas grown in Navarre, a local company (Grupo AN) provided data on electricity consumption and other production costs associated with the shelling and milling processes. During these processes, a 12% weight loss was recorded due to waste and pea hulls.

Notes:

Image of High Hydrostatic Pressures machinery of the Experimental design flow chard. Available online: https://www.smartchain-platform.eu/de/innovation/high-hydrostatic-pressure (accessed on 12 November 2024).

Image of Sous-vide machinery of the Experimental design flow chard. Available online: https://frigeriahosteleria.com/cocedor-sous-vide/10165-maquina-coccion-al-vacio-25-litros-sousvide-25.html (accessed on 12 November 2024).
